# An Experimental Study on the Shear Hysteresis and Energy Dissipation of the Steel Frame with a Trapezoidal-Corrugated Steel Plate

**DOI:** 10.3390/ma10030261

**Published:** 2017-03-06

**Authors:** Sudeok Shon, Mina Yoo, Seungjae Lee

**Affiliations:** 1 Department of Architectural Engineering, Korea University of Technology and Education, Cheonan 31253, Korea; sdshon@koreatech.ac.kr; 2 Interdisciplinary Program in Creative Engineering, Korea University of Technology and Education, Cheonan 31253, Korea; yoomina@koreatech.ac.kr

**Keywords:** steel frame, trapezoidal-corrugated steel plate, shear wall system, hysteresis behavior, energy dissipation, cyclic loading test, shear buckling

## Abstract

The steel frame reinforced with steel shear wall is a lateral load resisting system and has higher strength and shear performance than the concrete shear wall system. Especially, using corrugated steel plates in these shear wall systems improves out-of-plane stiffness and flexibility in the deformation along the corrugation. In this paper, a cyclic loading test of this steel frame reinforced with trapezoidal-corrugated steel plate was performed to evaluate the structural performance. The hysteresis behavior and the energy dissipation capacity of the steel frame were also compared according to the corrugated direction of the plate. For the test, one simple frame model without the wall and two frame models reinforced with the plate are considered and designed. The test results showed that the model reinforced with the corrugated steel plate had a greater accumulated energy dissipation capacity than the experimental result of the non-reinforced model. Furthermore, the energy dissipation curves of two reinforced frame models, which have different corrugated directions, produced similar results.

## 1. Introduction

Various attempts have been made to improve seismic performance in the domain of conventional seismic design and reinforcement methods for resisting lateral forces, such as wind loads and seismic loads in building structures. These attempts include reinforcing with a steel plate, a steel brace or with an increased section and the addition of a new element. Among these, the steel plate shear wall is known as an excellent lateral-force-resisting system because it can utilize a thin steel plate, reduce its own weight and improve seismic resistance. Steel plates with high rigidity and ductility are widely used in the United States and Japan.

Early studies have been performed on potential methods of preventing out-of-plane buckling before shear yielding occurs and of increasing the stiffness and strength of buildings by using relatively thick steel plates or steel plates reinforced with vertical and horizontal stiffeners [1]. Studies focused on the post-buckling strength of steel plates that had been subjected to shear force have been carried out since 1980 from a different viewpoint from that of recent studies. The shear walls of steel plates without a reinforcement material have the advantage of resisting lateral force by tension field action arising from shear buckling before shear yielding occurs and of reducing the construction and construction costs [2,3]. Given the absence of reinforcement, however, such high stiffness can be obtained through the tension field action, but shear buckling occurs even under a low load, which again causes damages in the non-structural members when a minor earthquake occurs. When the steel plate is thicker than the design load, in particular, excess force is generated, thereby making it necessary to enlarge the columns and beams, which again leads to the ballooning of construction costs. Studies have been conducted recently on a steel plate shear system that does not use reinforcement materials and does not cause shear buckling under a low load [4]. Typically, there is a steel plate shear wall with a regular pattern of circular holes, a vertical slit, low yield strength and/or a partially-connected steel plate [4,5]. A corrugated steel plate has also been applied to a shear wall system [6,7].

Many studies have been conducted on corrugated steel plates and their application. In 1925, a corrugated steel web was proposed for structures for the first time [8], and in 1969, a pioneering study on shear behavior by Easley and McFarland [9] was published. Afterwards, more studies were conducted on the shear performance of these plates, with the majority of them focused on suggesting the equations required for calculating the shear buckling stress of plate girders with the corrugated plates. Such studies were followed by studies on the shear stiffness of the corrugation shape by analyzing bridges and girders [6,10,11,12,13,14,15,16,17,18,19], as well as by studies on lateral buckling, shear design, optimization of corrugated web beam and buckling tendency [20,21,22,23,24,25,26,27,28].

**Figure 1:**
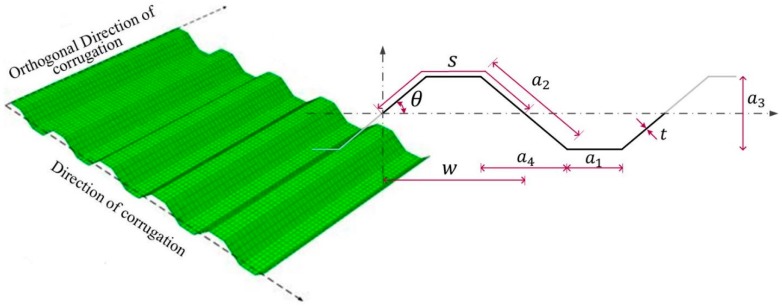
Shape parameter of a trapezoidal-corrugated steel plate.

Recently, the structural performance of the corrugated steel plate shear wall was evaluated, employing a lateral cyclic loading test [6]. Various types of corrugated steel plates have been attracting the attention of many researchers [5,7,29,30]. When corrugated steel plate is used in the shear wall system instead of a flat plate, it offers many advantages. Corrugation of the steel plate plays the role of a vertical stiffener and improves the plate’s out-of-plane rigidity. Moreover, as the corrugated steel plate can be designed as a thin steel plate, it is economically advantageous [30]. In particular, the corrugated steel plate can be selectively stiffened in the design owing to the accordion effect. The rigidity of the steel plate along the corrugation direction (see Figure 1) does not increase due to the flexibility in the deformation along the direction, thereby increasing the shear rigidity of the steel plate to a level much higher than that of the flat plate [31].

In seismic design, the method of increasing the member size or adding braces is used occasionally to improve the seismic performance of a structure, but this method has some disadvantageous effects. Another method is to use an energy dissipation device to dissipate the input energy via various mechanisms (e.g., friction, metallic yielding, viscosity, viscoelasticity) [32]. The study on dissipation capacity has been performed in the study of steel frame reinforced with corrugated steel plate, as well [6], and a specimen composed of a corrugated plate of t=1.25 mm fixed on the fish plate with bolts was used in this study. The corrugation shape used for the study was θ=30°, and both $a_{1}$ and $a_{2}$ shared the same shape with an identical size. However, the corrugated plate demonstrated a different buckling tendency and dissipation capacity depending on the corrugation shape and boundary condition [26,27,30], and experimental studies on the dissipation characteristics of such corrugated plates have not been sufficiently performed.

In this paper, attention is focused on a shear wall steel frame reinforced with a corrugated plate that was welded to the surrounding frames. An experimental study was conducted to evaluate cyclic behavior and energy dissipation of the test specimens. The test specimen was designed by changing the direction of corrugation in the shape, where the corrugation angle θ is 30° and $a_{1}\neq a_{2}$ (see Figure 1 and Table 1), and the specimen’s structural characteristics were also analyzed in the cyclic loading test. In general, to fully mobilize the local shear buckling strength, Lindner and Huang [33] suggest that the angle (θ) should not be less than 30°. The contents of the paper are shown below. In Section 2, the design of the test specimen, the shape determination and the cyclic loading test and test methods are described. In Section 3, the test results, including the load-displacement relationship, strain and energy dissipation capacity are compared, and the conclusion is presented in Section 4.

## 2. Cyclic Loading Test

### 2.1. Test Specimen Design

**Figure 2:**
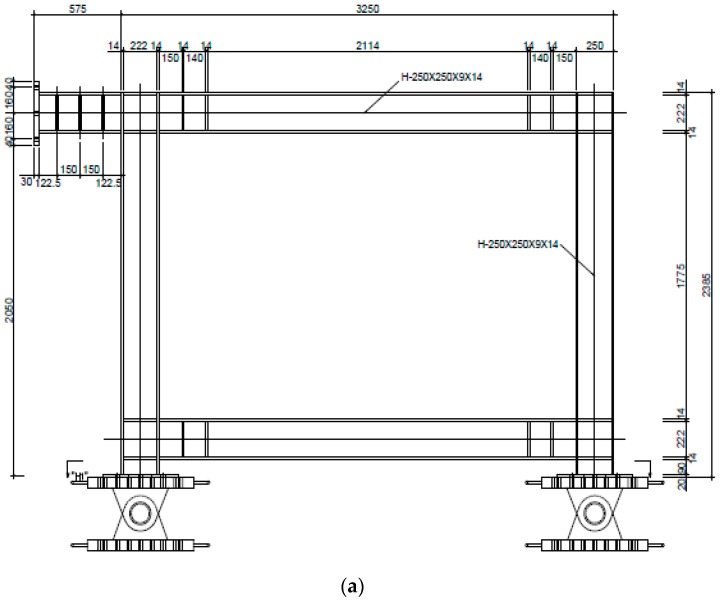

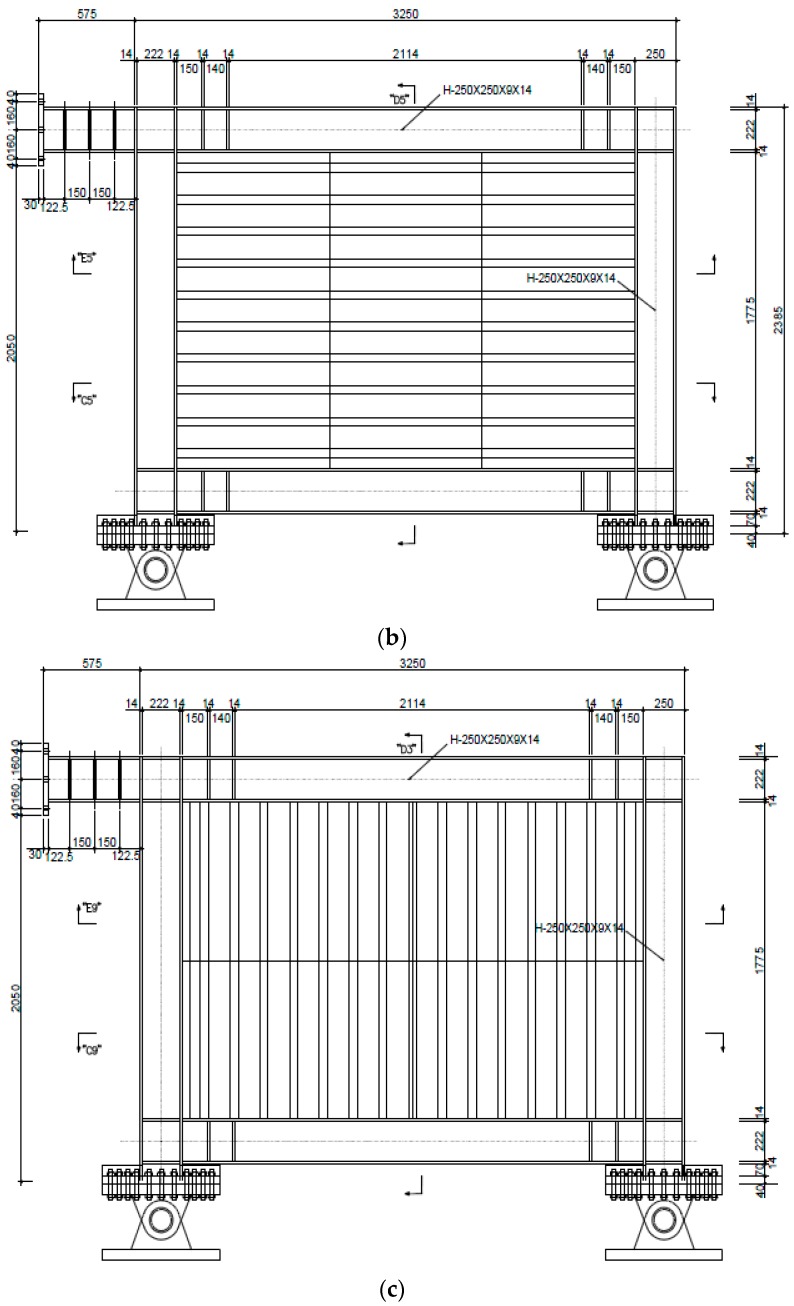
Configuration of the test specimens (unit: mm): (**a**) Simple steel frame: FR-00; (**b**) Steel frame model with a vertical corrugation: FR-TR-V; (**c**) Steel frame model with a horizontal corrugation: FR-TR-H.

This test was designed by considering two models as test specimens: a framed model (FR-00) without a steel plate and a model employing a trapezoidal-corrugated steel plate, as shown in Figure 2 and Table 1. The framed test specimen that was applied with the corrugated steel plate was also designed in two models (FR-TR-V and FR-TR-H): one with a vertical corrugation direction and the other with a horizontal corrugation direction. As shown in the figure, FR-00 in Figure 2a is the test specimen with only a basic frame while FR-TR-V and FR-TR-H in Figure 2b, c are the test specimens that were made by reinforcing FR-00 with a corrugated steel plate.

The corrugated steel plate that was applied to the test specimen had horizontal or vertical panels, and the main parameters of the applied members are shown in Table 1. As shown in the table, the corrugated steel plate that was used in the test specimen was a plate with the following dimensions: $a_{1}$ = 48 mm; $a_{3}$ = 75 mm; θ = 30°; and t = 3.2 mm. The FR-TR-V was manufactured by welding three pieces of H = 917 mm corrugated plate. The FR-TR-H was also manufactured by welding four pieces of H = 888 mm with the same corrugation shape, but with their direction rotated by 90°. The steel used for the corrugated plate was made of the equivalent grade steel pursuant to SS400 grade of the Korea Standard D 3503 (KS D 3503) [34], and the frame was made of the equivalent grade steel pursuant to SM400A grade of the Korea Standard D 3515 (KS D 3515) [35]. In the specimen, the size of the H-section steel beam is 250 × 250 × 9.0 × 14, as shown in Table 1, and the ends of the beams were reinforced with stiffeners (120.5 × 222), as shown in Figure 2.

### 2.2. Cyclic Loading and Measurement

**Figure 3:**
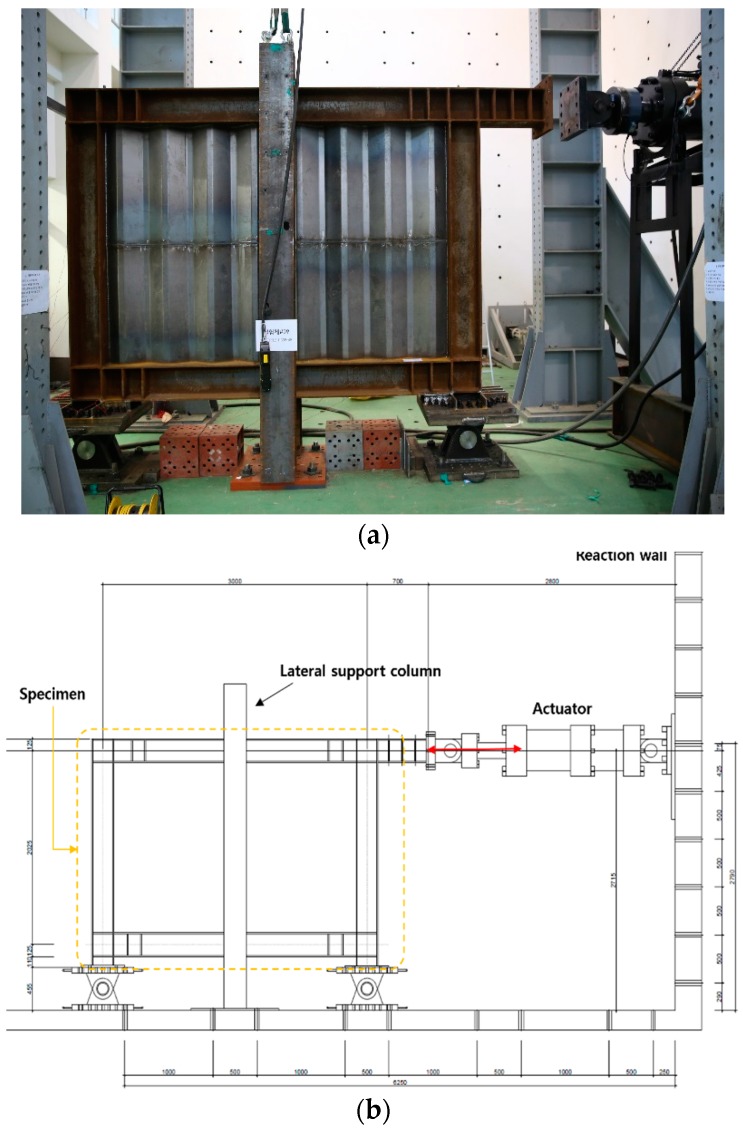

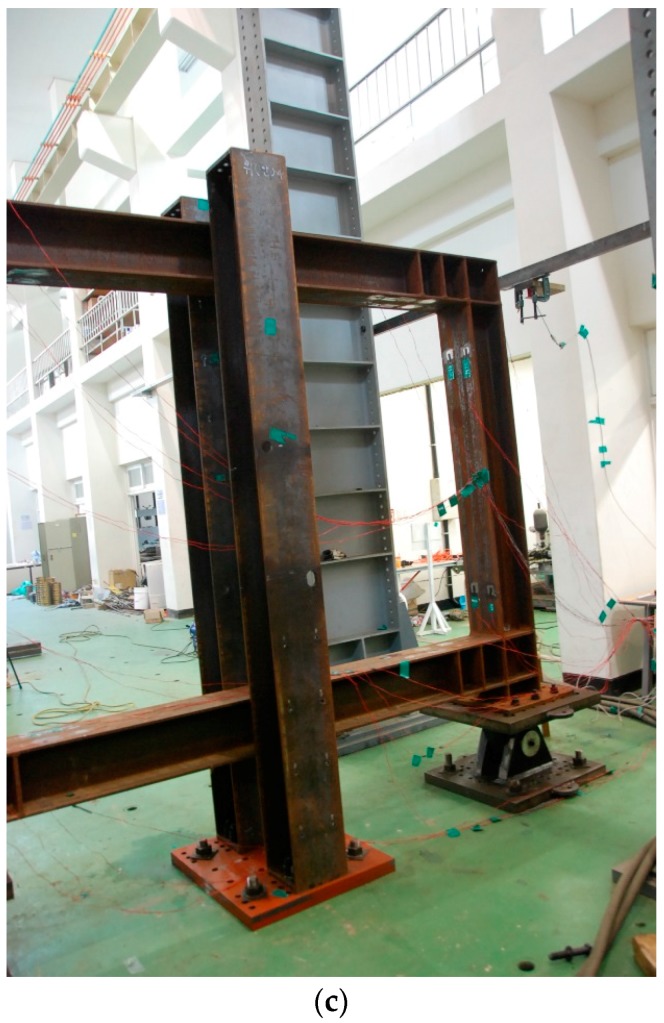
Experimental arrangement: (**a**) setup of specimens; (**b**) detail of the cyclic loading system; (**c**) two H-section steel beams.

The installation conditions of the test jig are shown in Figure 3. The test jig was installed as shown in Figure 3a, and its design is shown in Figure 3b. A 2000-kN-class device was used as an actuator, which was then supported by the reaction wall, and the loading was applied to the end of the upper beam. Two hinges were installed on the lower part to suppress the bending-deformation of the test specimen, and two H-section steel beams, as shown in Figure 3a,c, were installed on the front and back side of the test specimen to suppress the out-of-plane deformation of the specimen.

**Figure 4:**
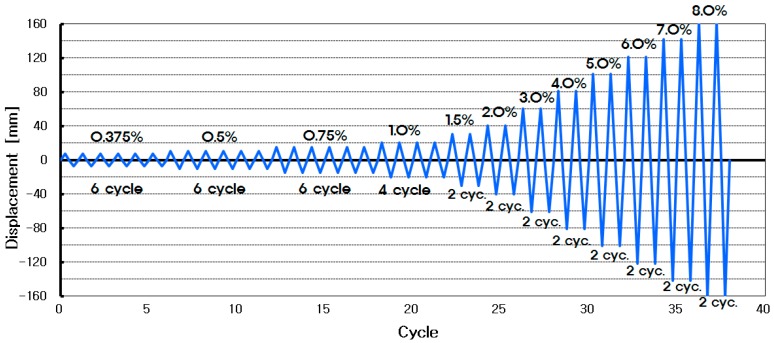
Displacement-controlled loading sequence.

The loading sequence is shown in Figure 4, with the loading applied via displacement control. The displacement control was realized according to the load-carrying capacity based on the drift ratio of the test specimens, while the measurement was carried out using the directly-measured values, with the loading device and the wire displacement meter installed on the left side of the upper rigid-body block.

### 2.3. Strain Measurement

**Figure 5:**
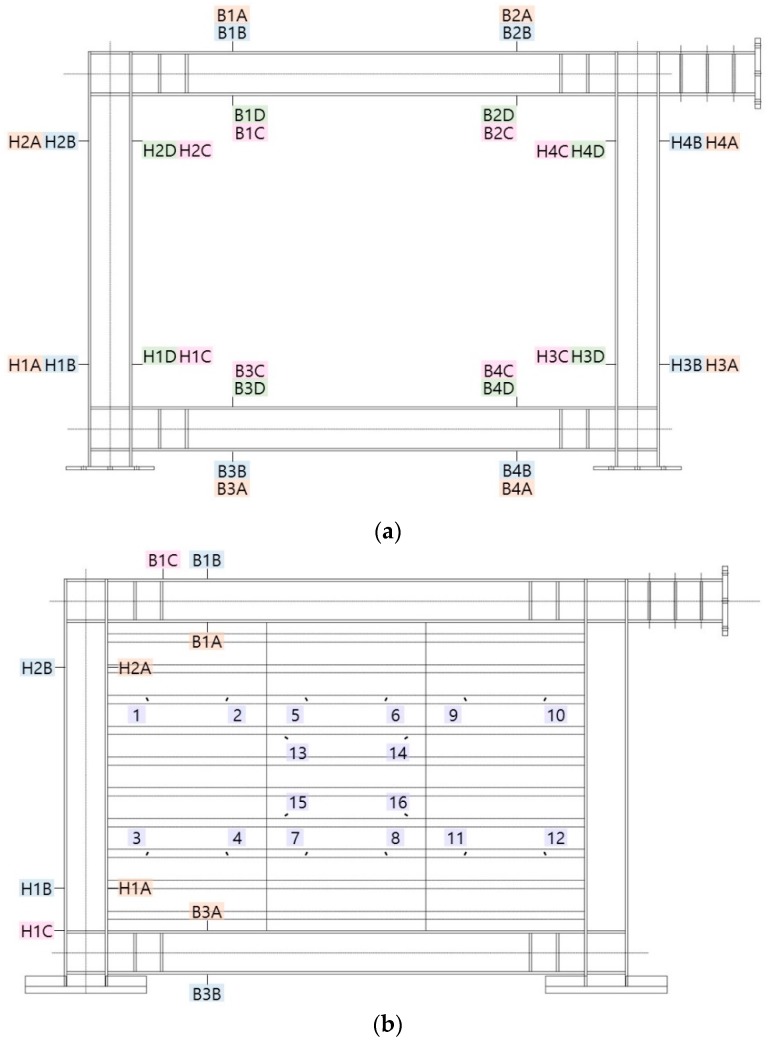

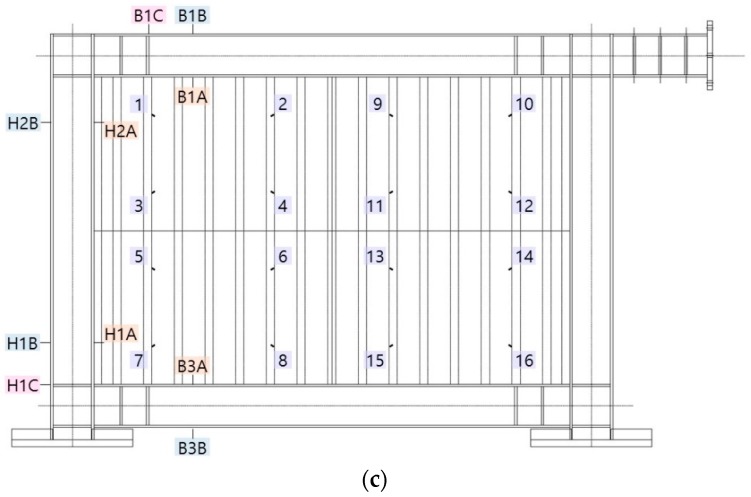
Location of strain gages: (**a**) FR-00; (**b**) FR-TR-V; (**c**) FR-TR-H.

To analyze the hysteresis behavior of the test specimens, strain gauges were attached to the ends of the columns and beams, as well as to the center and both ends of the corrugated steel plates. The strain gauges were attached along the diagonal directions of the braces and gusset plates. The location of each test specimen is described in detail below. The strain gauges of FR-00 were located as shown in Figure 5a; H1A(C) and H1B(D) were located on the lower end of the left column, H2A(C) and H2B(D) on the upper end of the left column, H3A(C) and H3B(D) on the lower end of the right column, H4A(C) and H4B(D) on the upper end of the right column, B1A(C) and B1B(D) on the left side of the upper beam, B2A(C) and B2B(D) on the right side of the upper beam, B3A(C) and B3B(D) on the left side of the lower beam and B4A(C) and B4B(D) on the right side of the lower beam. The FR-TR-V and the FR-TR-H were located as shown in Figure 5b,c: H1B(A) and H1C on the lower end of the left column, H2B(A) at the upper end of the left column, B1B(A) and B1C on the left side of the upper beam, B3B(A) on the left side of the lower beam and Gauges 1–16 along the diagonal direction on the corrugated steel plate.

### 2.4. Theoretical Values of the Test Specimens

**Figure 6:**
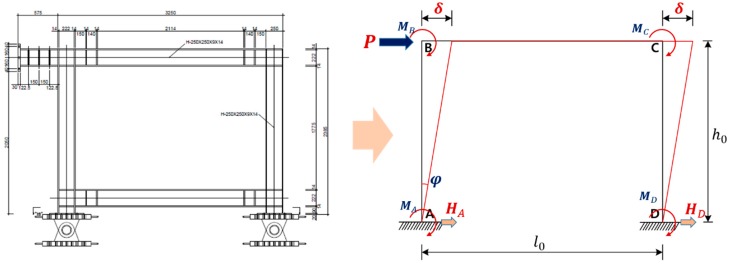
A simplified model of a steel frame specimen.

Theoretical values of the initial stiffness (Kia), plastic moment ($M_{p}$) and collapse load ($P_{c}$) of the steel frame specimen can be calculated using the simplified model of the specimen as shown in Figure 6. Here, the simplified model is assumed to be a steel frame with a beam length ($l_{0}$) of 3 m and a column height ($h_{0}$) of 2.025 m.

The equilibrium condition at Nodes B and C is obtained using the slope deflection equation of each member; where EI, $\theta_{B\left(or\;C\right)}$ and φ are the flexural rigidity of the element, the rotational angles of the node B (or C) and a chord rotation, respectively.

(1) $$\sum^{\text{​}}M_{\left(at\;B\right)}=\frac{2EI}{h_{0}}\left(2\theta_{B}-3\varphi \right)+\frac{2EI}{l_{0}}\left(2\theta_{B}+\theta_{C}\right)=0\;\sum^{\text{​}}M_{\left(at\;C\right)}=\frac{2EI}{h_{0}}\left(\theta_{B}+2\theta_{C}\right)+\frac{2EI}{l_{0}}\left(2\theta_{B}-3\varphi \right)=0$$

The sum of the horizontal reactions $H_{A}$ and $H_{D}$ (see Figure 6) of the simplified frame model is equal to the external load P, and the following equation can be obtained using the shear equilibrium condition.

(2) $$P=\frac{2EI}{l_{0}^{2}}\left(3\theta_{B}+3\theta_{C}-12\varphi \right)$$

When Equations (1) and (2) are solved as simultaneous equations assuming P=1 kN, $\theta_{B}$, $\theta_{C}$ and φ are as shown below.

(3) $$\theta_{B}=\theta_{C}=9.17\times 10^{-5}\;\varphi =\frac{\delta }{h_{0}}=1.23\times 10^{-4}$$

Therefore, Kia, $M_{p}$ and $P_{c}$ are able to be calculated by using Equation (3) and they are as shown below.

(4) Kia=Mφ=16,462 kN⋅m/radMp=σy×Z=312.3 kN⋅mPc=4Mph0=616.9 kN

The theoretical values of the simplified model discussed above are compared with the experiment results that will be discussed in the next section.

### 2.5. Elastic Shear Buckling of the Corrugated Plate

Buckling strength and buckling mode for elastic shear buckling of the corrugated plates can be calculated through theoretical equations before designing test specimens. In general, the shear buckling of a corrugated plate is represented by local buckling, global buckling and interactive buckling, respectively. The equations for each buckling strength are given below in Equations (5)–(7) [26,27,30].

(5) $$\tau_{cr,L}=\kappa_{L}\frac{\pi^{2}E}{12\left(1-\nu^{2}\right)}{\left(\frac{t}{a}\right)}^{2}$$

(6) $$\tau_{cr,G}=\kappa_{G}\frac{\sqrt[{4}]{D_{x}D_{y}^{3}}}{th^{2}}$$

(7) $$\frac{1}{\tau_{cr,I}^{2}}=\frac{1}{\tau_{cr,L}^{2}}+\frac{1}{\tau_{cr,G}^{2}}$$

where $\tau_{cr,L}$, $\tau_{cr,G}$ and $\tau_{cr,I}$ are elastic shear buckling strength for a local, a global and an interactive buckling, respectively. $\kappa_{L\left(or\;G\right)}$ refers to a local (or global) buckling factor. h is the length of a orthogonal direction of corrugation. a is the maximum width of the corrugated panel ($=\max\left(a_{1},a_{2}\right)$), and $D_{x\left(or\;y\right)}$ is the flexural stiffness of a corrugated plate about the strong (or weak) axis.

Table 2 shows the buckling strength of corrugated plates applied to the test specimen using the equations discussed above. In order to investigate the buckling tendency, the ratio of the buckling stress G/L ($=\tau_{cr,G}/\tau_{cr,L}$) and a modification factor of global buckling strength $\psi_{G}$ [27], as well as the modified buckling strength $\tau_{cr}^{m}$ calculated using the above factors were calculated and shown together in Table 2.

Table 2 shows that both FR-TR-V and FR-TR-H models are less susceptible to local buckling and can predict global-typed interactive buckling. With regard to the buckling tendency, the range of interactive buckling was suggested as 0.12≤G/L≤2.45 in the literature of Shon et al. [26,27]. In particular, the global-typed interactive buckling mode can be expected as G/L approaches 0.12, and the theoretical value and the error of $\psi_{G}$ as calculated by Equations (5)–(7) also increase [27]. Therefore, the FR-TR-H model has larger $\psi_{G}$ than the FR-TR-V model, and the buckling mode can also predict a global buckling. The modified buckling strengthes ($\tau_{cr}^{m}$) of the FR-TR-V and FR-TR-H specimens are 2.324$\;\times 10^{5}\;\mathrm{kN}/m^{2}$ and 1.940 $\;\times 10^{5}\;\mathrm{kN}/m^{2}$, respectively, and the strength of the FR-TR-V model is about 19% larger than that of FR-TR-H. Given that $\tau_{cr}^{m}$ is underestimated at the boundary of G/L, however, the difference between the two values will be smaller [27].

## 3. Analysis of the Test Results

### 3.1. Initial Stiffness and Collapse Load

In this section, the collapse load, fracture type, structural performance, hysteretic behavior characteristics and energy dissipation capacity of the structure according to the cyclic loading were investigated based on the test results of the specimen, with the initial stiffness and collapse load data shown in Table 3. Table 3 shows the initial stiffness (Kit) and maximum moment (Mmaxt) of each test specimen, which were obtained from the test data. The theoretical initial stiffness (Kia), plastic moment ($M_{p}$) and collapse load ($P_{c}$) of the frame in the table were summarized for the purpose of comparison, as shown in Section 2.4.

### 3.2. Collapse Types of the Specimens

**Figure 7:**
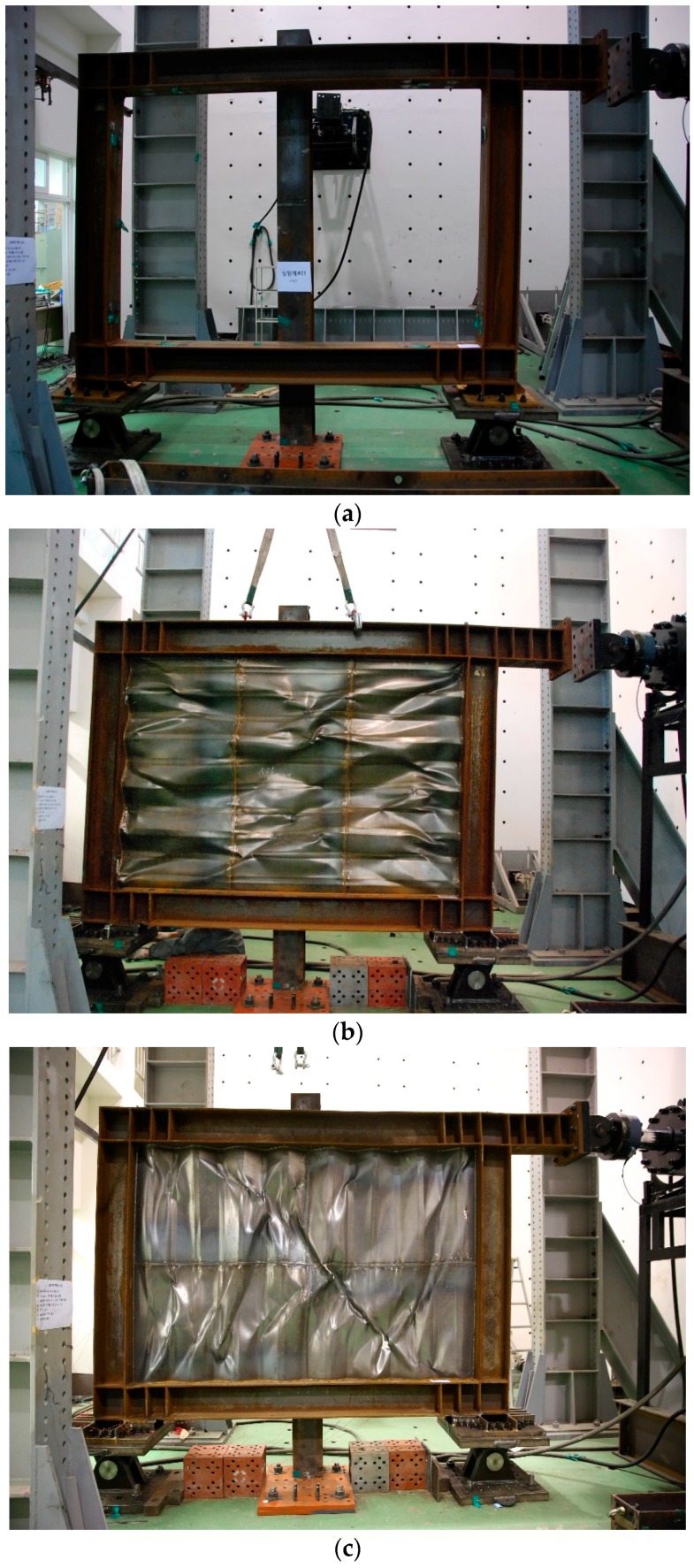
State of the specimens at ultimate stage: (**a**) FR-00; (**b**) FR-TR-V; (**c**) FR-TR-H.

The ultimate state of the test specimen was the fracture of the frame and shear buckling of the trapezoidal-corrugated steel plate. The ultimate stages of the specimens are shown in Figure 7, and their respective collapse types are shown below.

**Figure 8:**
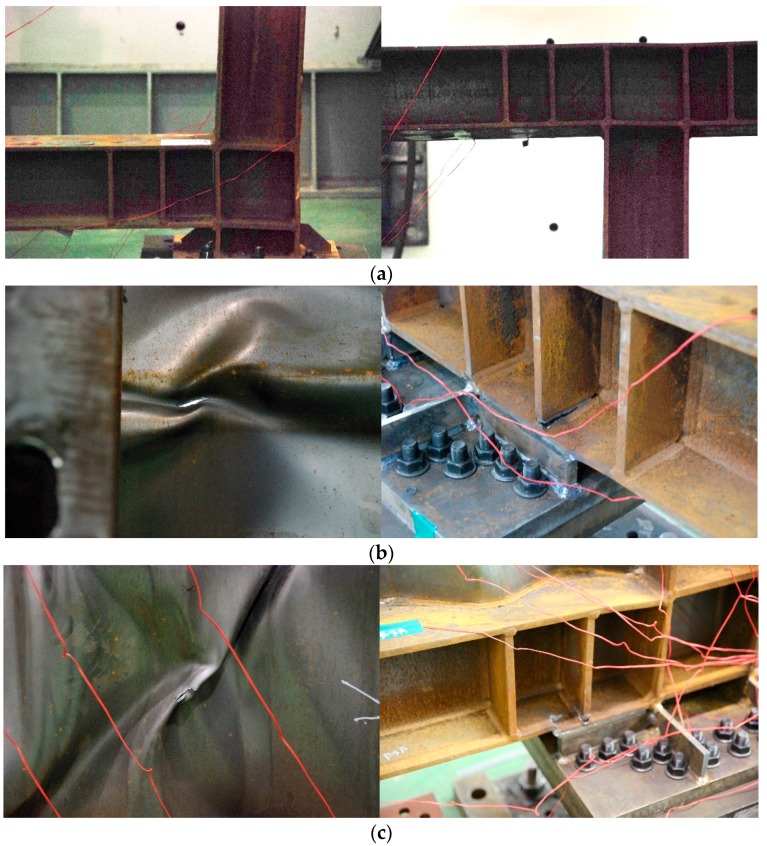
Observed failure location of the specimens: (**a**) FR-00; (**b**) FR-TR-V; (**c**) FR-TR-H.

In the case of FR-00, the maximum load was reached at a displacement of 162 mm (drift ratio: 8%) with a non-reinforced frame, as shown in Figure 7a, and buckling of the column-beam joint was visually observed after a 121-mm displacement (drift ratio: 6%). The FR-TR-V buckled at a 40.5-mm displacement (drift ratio: 2%) and reached the final state, as shown in Figure 7b, by reaching the maximum load at a displacement of 162 mm (drift ratio: 8%). The corrugated steel plate cracked at the position where the buckling field was inverted due to cyclic loading, as shown in Figure 8b. In the case of the frame, the stiffener and flange welds on the end of the lower beam were cracked (see Figure 8a). The FR-TR-H started to buckle at the 40.5 mm of maximum displacement (drift ratio: 2%) and reached the final state as shown in Figure 7c. As shown in Figure 8c, cracks occurred at the position where the buckling field was inverted by the cyclic loading of the corrugated steel plate, and the flange welds of the stiffener and the lower beam at the end of the lower beam were cracked. The fracture patterns of FR-TR-V and FR-TR-H were similar.

### 3.3. Load-Displacement Relationship

**Figure 9:**
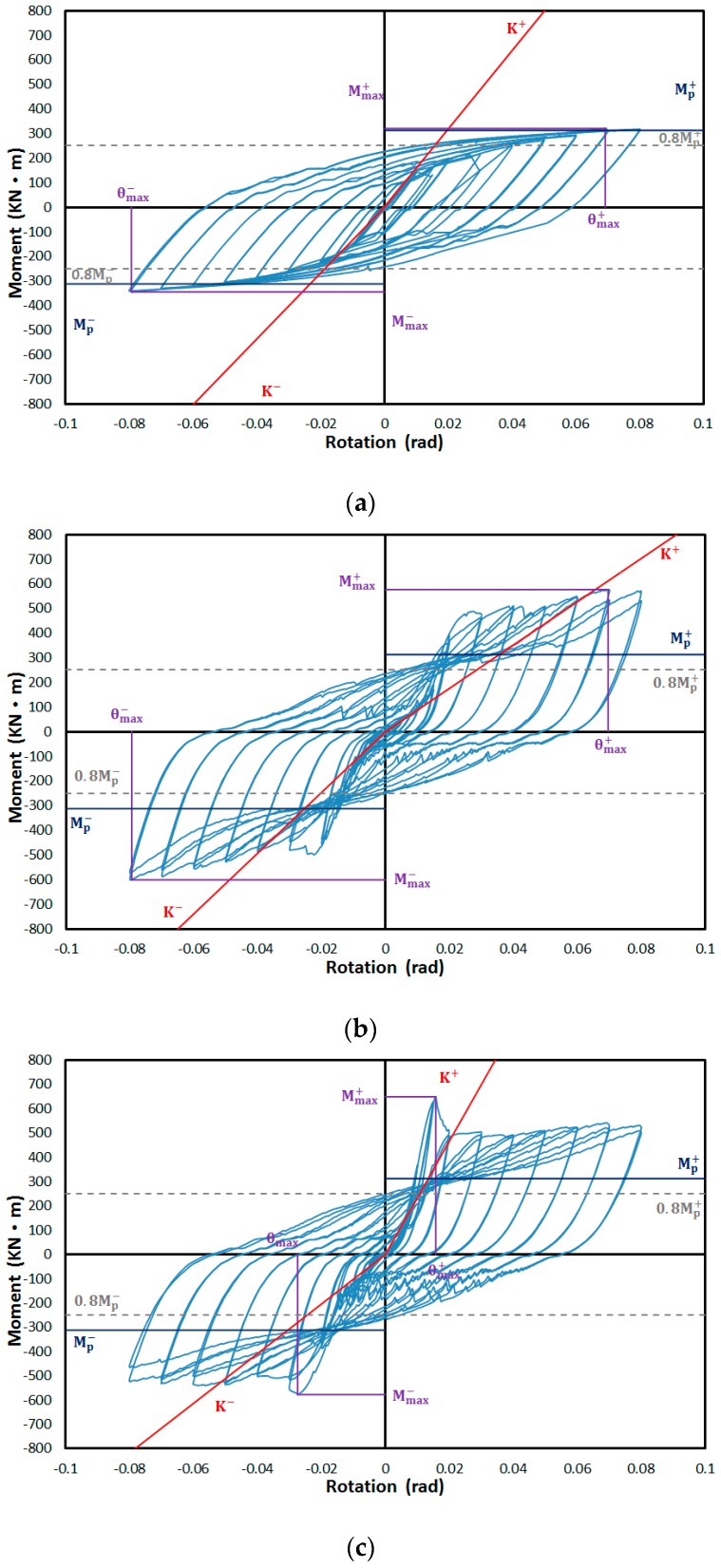
Hysteresis curves of the specimens: (**a**) FR-00; (**b**) FR-TR-V; (**c**) FR-TR-H.

In the test specimens that had been reinforced with corrugated steel plates, the columns and beams were plasticized after buckling of the corrugated steel plates, and the maximum strength was larger than that of the non-reinforced specimens. The structural performances of the two reinforced specimens were similar. The load displacement curve for each specimen is shown in Figure 9 and is analyzed as shown below.

The initial stiffness of FR-00 was 15,923 kN·m/rad in the positive loading direction and 13,391 kN·m/rad in the negative loading direction. The initial stiffness per the theoretical initial one was 96.74% (the positive loading direction) and 81.34% (the negative loading direction). The maximum moment was 320.5 kN·m in the positive loading direction and 345.4 kN·m in the negative loading direction, which were up to 102.6% and 110.6% of the theoretical plastic moment, respectively. The initial stiffness of the FR-TR-V was 8781 kN·m/rad in the positive loading direction and 12,319 kN·m/rad in the negative loading direction. The maximum moment was 575.9 kN·m in the positive loading direction and 601.2 kN·m in the negative loading direction, which were up to 184% and 192% of the theoretical plastic moment, respectively. The initial stiffness of FR-TR-H was 23,282 kN·m/rad in the positive loading direction and 10,236 kN·m/rad in the negative loading direction. The maximum moment was 648.5 kN·m in the positive loading direction and 577.3 kN·m in the negative loading direction, which were up to 207.7% and 184.9% of the theoretical value of the plastic moment, respectively. As mentioned earlier, the theoretical and test results of the FR-00 specimens were similar, and the maximum strength of the FR-TR-V and FR-TR-H specimens exceeded the structural performance of the FR-00 specimens.

### 3.4. Strain Curves

The plasticity of the columns and beams of the reinforced test specimen started later than that of FR-00, a non-reinforced specimen, and this was because the damage was concentrated on the corrugated steel plates at a small displacement level due to the reinforcement with the corrugated steel plates. The variation in the strain of each specimen is shown below.

**Figure 10:**
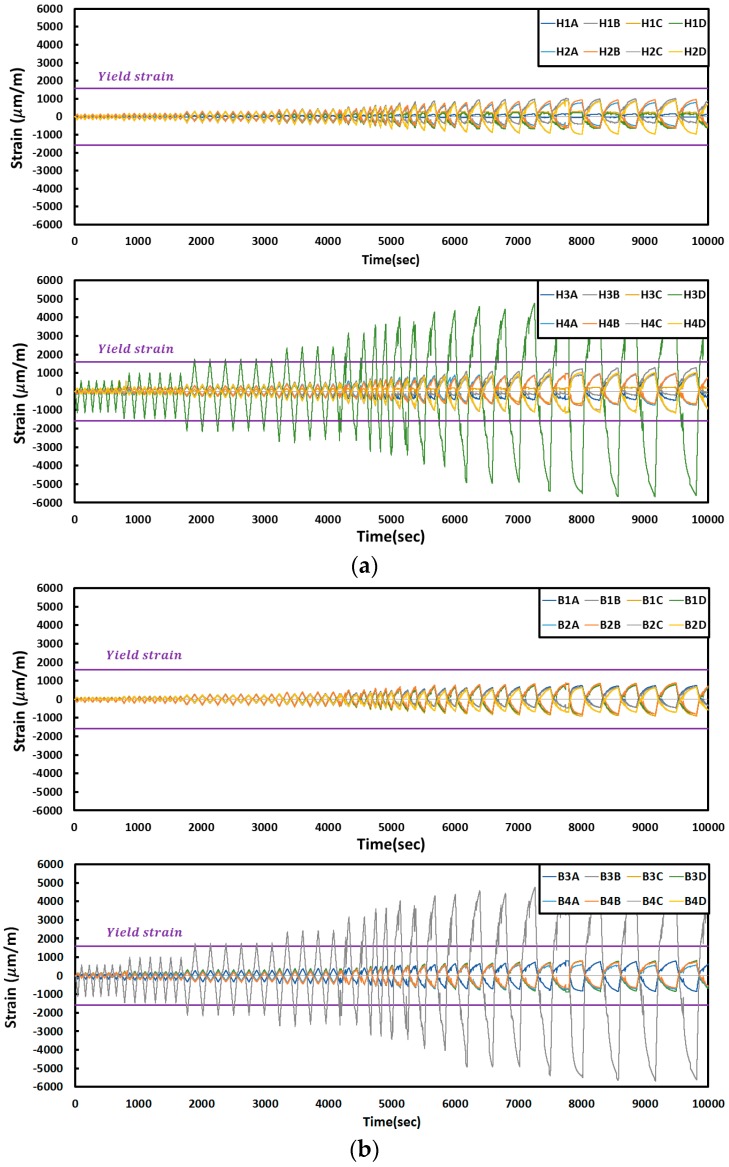
Strain curves of the specimens (FR-00): (**a**) column; (**b**) beam.

The changes in the strain according to the FR-00 test results are shown in Figure 10. The B3B gauge strain on the lower beam reached a maximum value of 2124 (μm/m) when displacement was 15.188 mm, thereby triggering plastic deformation. The strain gauges on the column showed similar amounts of strains among H1B, H1C and H1D on the left column, as well as among H2A, H2B, H2C and H2D. In addition, the amounts of strain at H3A and H3B on the right column were similar in the beginning, with comparable amounts of strain observed as well at H4A, H4B, H4C and H4D. The strain gauges on the beam displayed similar amounts of strain at B1A and B1B on the upper beam, while the strain values at B1C, B1D, B2A and B2B also changed in a similar fashion.

**Figure 11:**
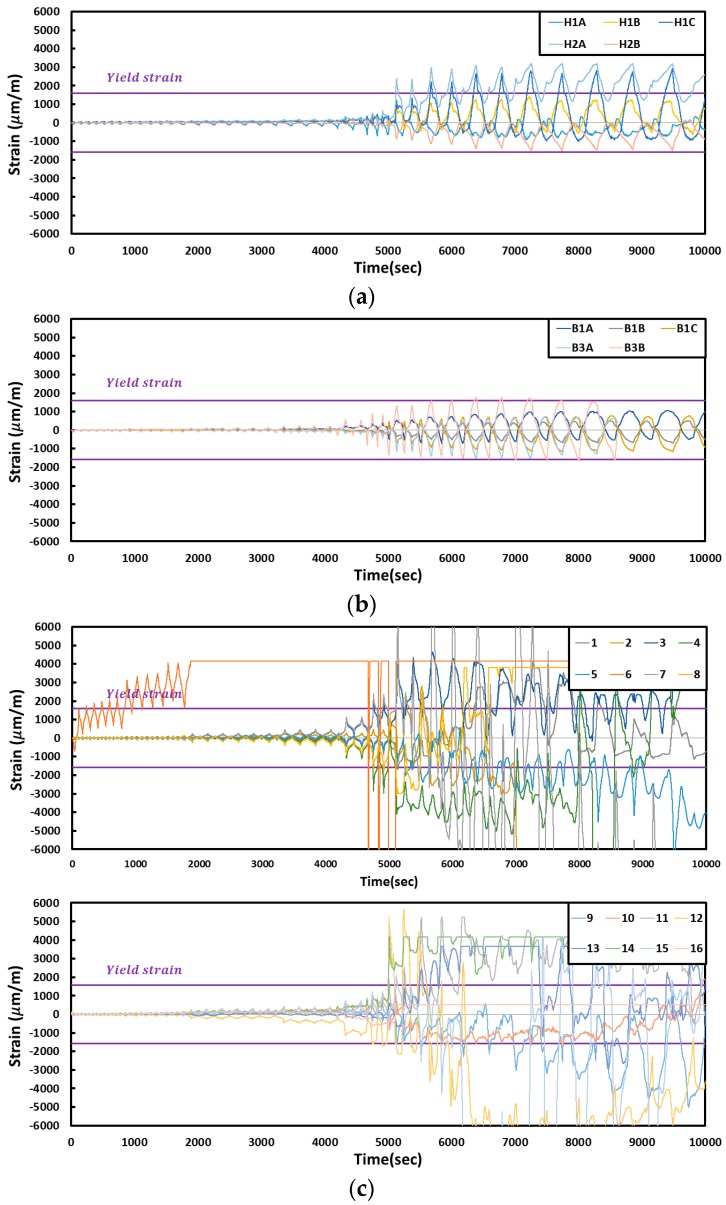
Strain curves of the specimens (FR-TR-V): (**a**) column; (**b**) beam; (**c**) plate.

The changes in the strain according to the FR-TR-V test results are shown in Figure 11. Local buckling in the inclined panel of the plate occurred at a displacement of 40.5 mm, and the strains at Gauges 3, 4, 7, 12 and 14 on the corrugated steel plate were 2095, 2340, 2411, 1719 and 4152 (μm/m), respectively. Local buckling occurred at the central part on the plate when the displacement was 60.75 mm, and plastic deformation occurred at Gauges 15, 9, 11, 2, 5, 8 and 1, each indicating 2125, 1589, 1823, 2473, 2020, 3060 and 1679 (μm/m), respectively. When displacement was 81 mm, the local buckling escalated to global buckling, thereby prompting buckling at the upper and lower parts with the remaining Gauges 13 and 10, each demonstrating strains of 2441 and 1537 (μm/m), respectively. The strain gauge on the column showed that the H2A gauge indicated a strain of 2378 (μm/m) at the 60.75-mm displacement, while the H1C gauge demonstrated a strain of 2214 (μm/m) at a displacement of 81 mm. The strain gauge on the beam showed that the B3B gauge demonstrated a strain of 1695 (μm/m) at a displacement of 81 mm.

**Figure 12:**
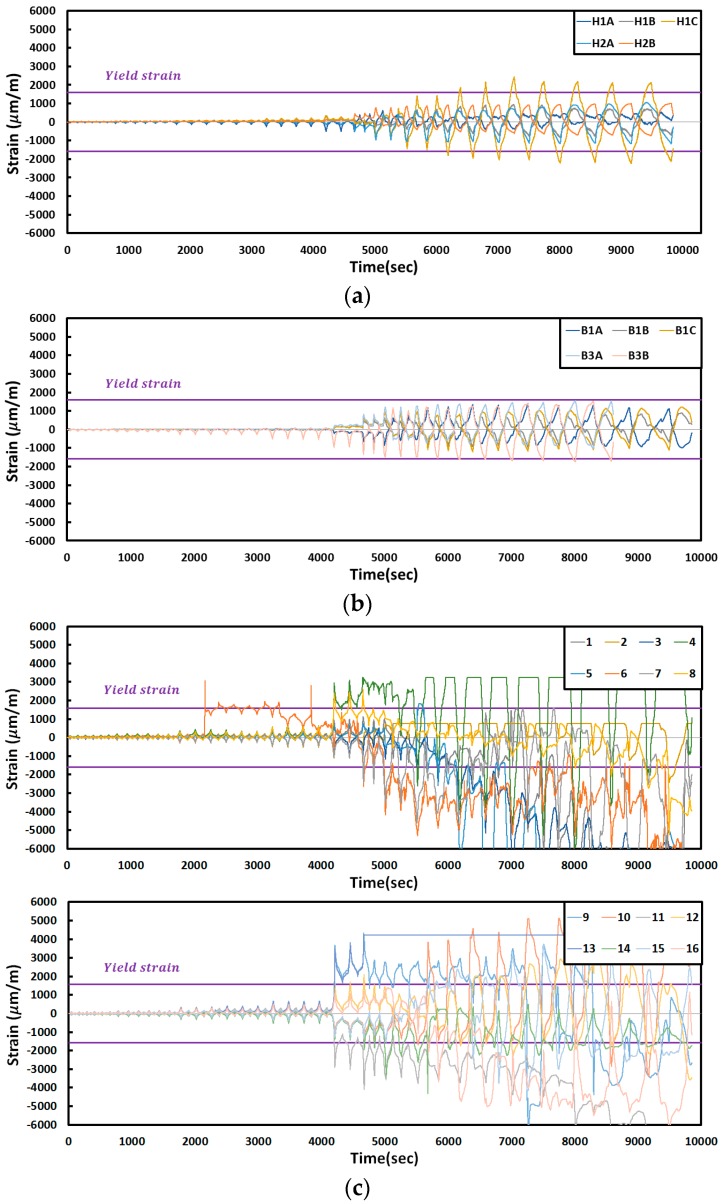
Strain curves of the specimens (FR-TR-H): (**a**) column; (**b**) beam; (**c**) plate.

The changes in the strain according to the FR-TR-H test results are shown in Figure 12. The strain that was observed in Gauge 6 on the corrugated steel plate was 3066 at a 15.188-mm displacement. When displacement was 30.375 mm, the strains that were observed at Gauges 9, 13, 11, 4, 8 and 12 were 3694, 3484, 2897, 2978, 2416 and 1659 (μm/m), respectively. When displacement was 40.5 mm, global buckling started at the center of the corrugated plate, and the strains at Gauges 10, 14 and 7 were 1542, 1932 and 2406 (μm/m), respectively. Global buckling escalated at the 60.75-mm displacement, with Gauges 15 and 3 indicating strains 1537 and 1574 (μm/m). The strains at Gauges 1, 5 and 16 and at the B3B gauge were 1867, 1808, 2122 and 1556 (μm/m), respectively, when displacement was 81 mm. When displacement was 101.25 mm, the H1C gauge on the column indicated an 1816 (μm/m) strain.

### 3.5. Energy Dissipation Capability

**Figure 13:**
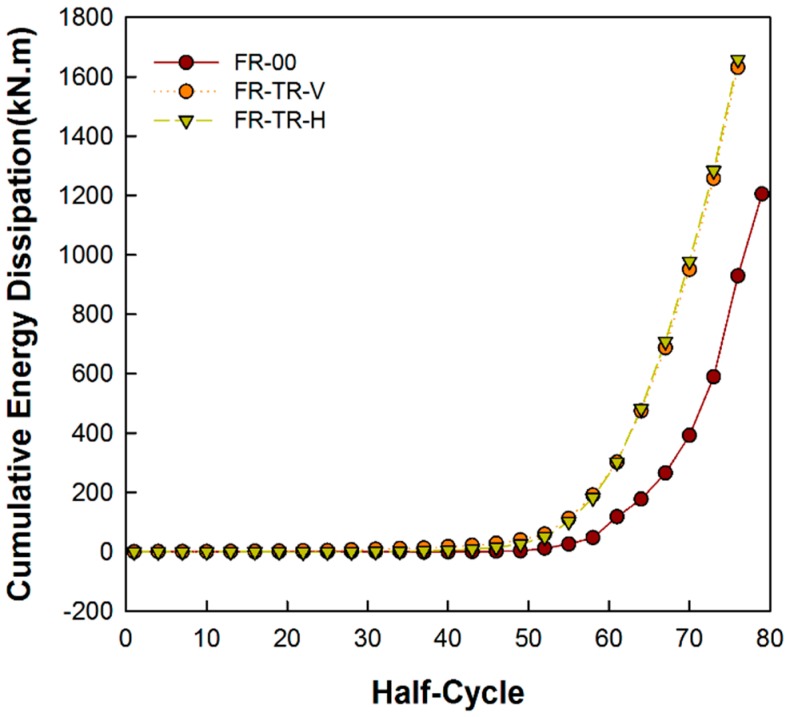
Cumulated energy dissipation curves of the specimens.

To evaluate how well the energy from the cyclic load is dissipated, the dissipated energy of the specimens were compared and analyzed. The dissipated energy of the FR-00 specimen, as well as that of the FR-TR-V and FR-TR-H specimens were compared to show the cumulated energies up to 37th cycle (final cycle) in Figure 13. The cumulated energy $E_{m}$ at the 30th cycle (5% drift) and 37th cycle (8% drift) of the cyclic load is as shown in Table 4.

As shown in the figure, the cumulative energy dissipation curves of FR-TR-V ($E_{V}$) and FR-TR-H ($E_{H}$) were similar, but were larger than that of the FR-00 ($E_{0}$). In the table, the energies $E_{V}$ and $E_{H}$ of the specimens at both 5% and 8% drift are larger than $E_{0}$. In the case of FR-TR-V specimens, the $E_{V}$ at both 5% and 8% is about 2.4- and 2.1-times greater than $E_{0}$, respectively. In the FR-TR-H specimen, the $E_{H}$ values are about 2.9- and 2.1-times greater, respectively. At 5% drift, $E_{H}$ is greater than $E_{V}$, but approaching 8%, $E_{V}$ and $E_{H}$ became similar. From the results of the collapse aspect and the strain measurement, the FR-TR-V and FR-TR-H specimens showed buckling of the corrugated steel plate, but the columns and beams remained within the elasticity range and reached the final state after buckling.

## 4. Conclusions

In the experimental study that was conducted for the paper, a cyclic loading test was performed on the frame reinforced with a trapezoidal-corrugated steel plate to analyze structural performance. The characteristics of the hysteresis behavior and the dissipated energy were compared.

Based on the comparison results, the proposed frame reinforced with a trapezoidal corrugated steel plate shows that its maximum strength is higher than that of the non-reinforced frame, and its cumulative dissipated energy was found to be larger than that of the non-reinforced frame. The input energy generated by the cyclic loading is dissipated via plastic deformation of the corrugated steel plate, and the corrugated steel plate buckled earlier than the frame did. From the experimental results, the maximum strength of reinforced frame test specimens is approximately 1.7–1.9-times higher than that of non-reinforced frame test specimens, and the cumulative dissipated energy is approximately 2.1-times higher than that of the non-reinforced frames. Judging from the experiment results of the reinforced test specimens, however, FR-TR-V has a maximum strength at 8.0% drift and FR-TR-H has a maximum strength at 2.0% drift. These results indicate that the structural performance and energy dissipation capacity of the corrugated steel frame are similar regardless of the corrugation direction, but the failure mechanism can be different depending on the corrugation direction. Experiment results show that the corrugated steel plate of the FR-TR-V test specimen experiences local buckling first, followed by global buckling, while the FR-TR-H corrugated steel plate has only showed global buckling. Judging from the calculation result of the ratio of the shear buckling (G/L), FR-TR-H is more likely to trigger global buckling than FR-TR-V with a different corrugation direction.

It has been reported that corrugated steel plates demonstrate significant differences in performance depending on their thickness, depth, length and shape. These differences are believed to vary in the case of frames to which corrugated steel plates with various shapes are applied. Further experimental and theoretical study considering corrugation shapes is necessary.

## Figures and Tables

**Table 1:** Details of the specimens.

Specimen	Column	Beam	H (t)	Parameters (See Figure 1)		
				$a_{1}$	$a_{3}$	θ
FR-00	H-250 × 250 × 9.0 × 14		-	-	-	-
FR-TR-V	H-250 × 250 × 9.0 × 14		917 (3.2)	48	75	30
FR-TR-H	H-250 × 250 × 9.0 × 14		888 (3.2)			

t: thickness of the corrugated plate (mm); $a_{1}$: horizontal length of the corrugated plate (mm); $a_{3}$: depth of the corrugation (mm); θ: inclined angle between panels (°).

**Table 2:** Shear buckling strength of corrugated plates of the specimens (unit: $\times 10^{5}\;\mathrm{kN}/m^{2}$).

Specimen	$\tau_{cr,L}$	$\tau_{cr,G}$	$\tau_{cr,I}$	G/L	$\psi_{G}$	$\tau_{cr}^{m}$
Corrugated plate of FR-TR-V	4.527	2.897	2.440	0.640	0.802	2.324
Corrugated plate of FR-TR-H	4.513	1.207	1.166	0.267	1.607	1.940

**Table 3:** Details of the specimens.

Specimen	Ki+t	Ki−t	Mmax+t	Mmax−t
	(kN⋅m/rad)	(kN⋅m/rad)	(kN⋅m)	(kN⋅m)
FR-00	15,923	13,391	320.5	345.4
FR-TR-V	8781	12,319	575.9	601.2
FR-TR-H	23,282	10,236	648.5	577.3

Ki+t, Ki−t: + (or −) dir. initial rigidity of the specimens; Mmax+t, Mmax−t: + (or −) dir. max. moment of the specimens; Kia: theoretical initial stiffness of the frame (=16,462 kN⋅m/rad); $M_{p}$: plastic moment of the frame (=312.3 kN⋅m); $P_{c}$: collapse load of the frame (=616.9 kN).

**Table 4:** Cumulated energy $E_{m}$ of the specimens.

Specimens	FR-00 ($E_{0}$) (kN⋅m)	FR-TR-V		FR-TR-H	
		$E_{V}$ (kN⋅m)	$E_{V}/E_{0}$	$E_{H}$ (kN⋅m)	$E_{H}/E_{0}$
$E_{m}$ at 5% drift	89.2	218.0	2.444	264.6	2.966
$E_{m}$ at 8% drift	599.5	1257.5	2.098	1283.8	2.141

$E_{m}$: Cumulative dissipated energy; where, *m* is the model name, e.g., the FR-00, FR-TR-V and FR-TR-H specimens are $E_{0}$, $E_{V}$ and $E_{H}$, respectively.
